# Sphingosine 1-Phosphate Receptor 2 and 3 Mediate Bone Marrow-Derived Monocyte/Macrophage Motility in Cholestatic Liver Injury in Mice

**DOI:** 10.1038/srep13423

**Published:** 2015-09-01

**Authors:** Le Yang, Zhen Han, Lei Tian, Ping Mai, Yuanyuan Zhang, Lin Wang, Liying Li

**Affiliations:** 1 Department of Cell Biology, Municipal Laboratory for Liver Protection and Regulation of Regeneration, Capital Medical University, Beijing 100069, China

## Abstract

Sphingosine 1-phosphate (S1P)/S1P receptor (S1PR) system has been implicated in the pathological process of liver injury. This study was designed to evaluate the effects of S1P/S1PR on bone marrow-derived monocyte/macrophage (BMM) migration in mouse models of cholestatic liver injury, and identify the signaling pathway underlying this process. S1PR_1–3_ expression in BMM was characterized by immunofluorescence, RT-PCR and Western blot. Cell migration was determined in Boyden chambers. *In vivo*, the chimera mice, which received BM transplants from EGFP-transgenic mice, received an operation of bile duct ligation (BDL) to induce liver injury with the administration of S1PR_2/3_ antagonists. The results showed that S1PR_1–3_ were all expressed in BMMs. S1P exerted a powerful migratory action on BMMs via S1PR_2_ and S1PR_3_. Furthermore, PTX and LY-294002 (PI3K inhibitor) prevented S1PR_2/3_-mediated BMM migration, and Rac1 activation by S1P was inhibited by JTE-013, CAY-10444 or LY294002. Administration of S1PR_2/3_ antagonists *in vivo* significantly reduced BMM recruitment in BDL-treated mice, and attenuated hepatic inflammation and fibrosis. In conclusion, S1P/S1PR_2/3_ system mediates BMM motility by PTX-PI3K-Rac1 signaling pathway, which provides new compelling information on the role of S1P/S1PR in liver injury and opens new perspectives for the pharmacological treatment of hepatic fibrosis.

Macrophages, the most plastic cells of the haematopoietic system, are found in all tissues and show great functional diversity. They play significant roles in development, homeostasis, tissue repair and immunity^1^. Kuppfer cells, resident macrophages in liver, are localized in the lumen of the liver sinusoids, and predominantly in the periportal area, derived from circulating monocytes. After liver injury, monocytes/macrophages are rapidly recruited to the liver; these cells have similar functional profiles to Kuppfer cells^2^. There is now considerable interest in the effects of bone marrow (BM)-derived cells on liver injury and repair. For example, multiple lines of evidence have indicated that after liver injury, numbers of BM-derived monocytes/macrophages (BMMs) migrate and accumulate at the sites of inflammation, therefore, play an important role in liver regeneration, remodeling of ECM, inflammation and fibrogenesis^3^,^4^,^5^,^6^. Recently it has been reported that the inflammation and fibrosis of injured liver were ameliorated after macrophages were depleted^7^. Our previous study has also demonstrated that reducing the recruitment of BMMs can attenuate hepatic inflammation and fibrosis in mouse models of bile duct ligation (BDL)- or carbon tetrachloride (CCl_4_)-induced liver injury^8^.

Sphingolipid metabolite sphingosine 1-phosphate (S1P) is one of the most important bioactive lysophospholipids. The numerous biological functions of S1P include regulation of cellular survival, proliferation, migration, differentiation, angiogenesis and vascular integrity, as well as the control of immunity^9^,^10^,^11^,^12^,^13^. Many of the actions of S1P in innate and adaptive immunity are mediated by its binding to five specific G protein-coupled receptors, designated S1P receptor type 1-5 (S1PR_1–5_). Recently S1P/S1PR system has emerged as a crucial regulator of immunity, and the control of immune cell trafficking is one of the hallmarks of the involvement of S1P/S1PR in a broad range of inflammatory diseases^14^,^15^. For example, some studies have documented the role of S1P/S1PR in chemotaxis of bone marrow cell population, such as T cells, mast cells and dendritic cells^16^,^17^,^18^. However, there are few studies demonstrating the effect of S1P/S1PR on BMM motility. Therefore, in this study we designed to evaluate the effects of S1P/S1PR on the migration of BMMs *in vitro* and in mouse models of cholestatic liver injury, and identify the signaling pathway underlying this process.

The phosphoinositide 3-kinase (PI3K) and their downstream Rac is believed to play a major role in regulating cells migration^19^,^20^. The small G protein Rac is one of the main regulatory factors involved in the reassembly of the actin cytoskeleton, which plays important roles in coordinating cell migration^21^,^22^,^23^. However, whether PI3K and Rac are involved in S1PR-mediated BMM migration remains largely unexplored. Therefore, the present study focuses on the effects of PI3K and Rac signals on S1PR-mediated BMM migration.

In this study, we first investigated the effects of S1P on BMM migration *in vitro*, characterized the S1PR subtypes which were implicated in this process, and the underlying signaling pathways. In addition, we determined the effect of S1PR on BMM recruitment in mouse models of BDL-induced liver injury. We reported that S1PR_2_ and S1PR_3_ mediate S1P-induced BMM migration *in vitro*, and G_i/o_, PI3K and Rac1 signals were involved in this process. *In vivo*, administration of S1PR_2/3_ antagonist significantly reduced BMM recruitment in BDL-treated mice as the proportion of BMMs decreased markedly compared with BDL group. Furthermore, inhibition of S1PR_2_ or S1PR_3_ decreased the generation of inflammatory cytokines, and attenuated the degree of hepatic inflammation and fibrosis. Our data provides new compelling information on the role of S1P/S1PR in cholestatic liver injury and opens new perspectives for the pharmacological treatment of hepatic fibrosis.

## Results

### S1P exerts a powerful pro-migratory action on BMM via S1PR_2_ and S1PR_3_

**Figure 1:**
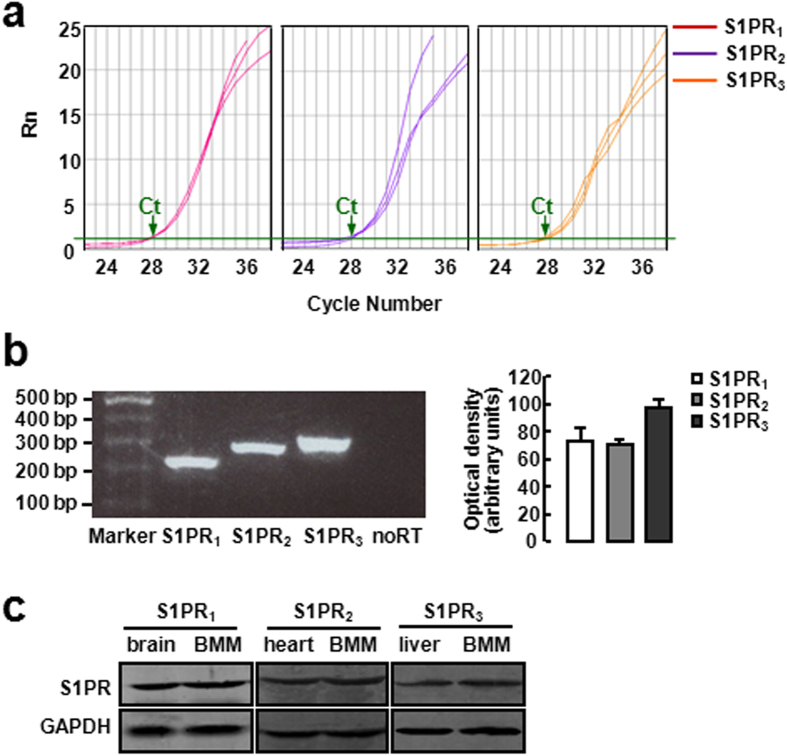
S1PR_1_, S1PR_2_ and S1PR_3_ are expressed in BMMs. (**a**) The amplification plot of S1PR_1–3_ expression in BMMs by real time RT-PCR. (**b**) The PCR products were size-fractionated in a 2% agarose gel. (**c**) Western blot analysis for protein expression of S1PR_1–3_ in BMMs. Brain, heart and liver tissue were used as positive controls.

We first examined the expression of S1PRs in BMM using real time RT-PCR and Western blot. As shown in the amplification plot in Fig. 1a, there were abundant expression of S1PR_1_, S1PR_2_ and S1PR_3_ in BMM, no different amount of each S1PRs. And then the PCR products were size-fractionated in a 2% agarose gel, showing that S1PR_1_, S1PR_2_ and S1PR_3_ were all detectable in BMM at the mRNA level (Fig. 1b). In addition, Western blot analysis showed the expression of S1PR_1_, S1PR_2_ and S1PR_3_ in BMM at the protein level, with brain, heart and liver tissue as positive controls, respectively (Fig. 1c).

**Figure 2:**
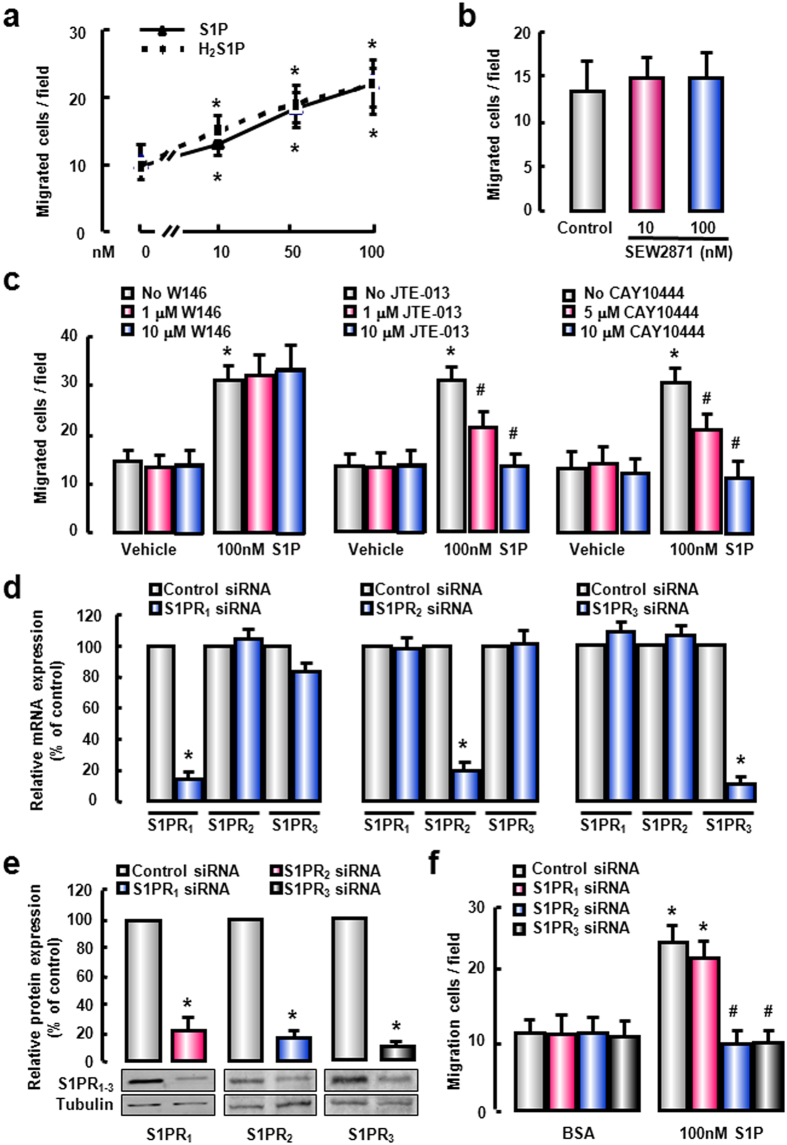
S1P induces the migration of BMMs via S1PR_2_ and S1PR_3_. (**a**) Serum-starved BMMs were allowed to migration for 4 hours in the presence of varying concentrations of S1P or H_2_S1P as indicated. Serum-starved cells were pretreated with S1PR_1_ agonist (**b**) or S1PR_1–3_ antagonists (**c**) for 1 hour before migration essay. Knock-down of S1PR_1–3_ mRNA (**d**) or protein (**e**) by S1PR_1–3_-siRNA transfection in BMMs. (**f**) Effects of S1PR_1_-, S1PR_2_- or S1PR_3_-siRNA on BMM migration in response to S1P. All results were confirmed in three independent experiments at least. *P < 0.05 vs. untreated control cells. ^#^*P *< 0.05 vs. S1P-treated cells alone.

Since S1P/S1PR signaling plays a significant role in the migration of BM-derived cells to the damaged liver^8^,^24^, we then explored the chemotactic behavior of BMM in response to S1P, by performing an *in vitro* migration assay in the Boyden chamber. The results showed that S1P exerted a powerful pro-migratory action on BMM in a dose-dependent manner (Fig. 2a). Likewise, H_2_S1P, a structural analogue of S1P which can only mediate its effects through a surface bound S1PR, mimicked the effects of S1P on BMM migration (Fig. 2a), suggesting that S1P induces the migration of BMM via its cell surface receptors. Next we determined which S1PR subtypes were implicated in S1P-induced migration of BMM, by employing specific S1PR agonists and/or antagonists. Stimulation of SEW2871, a selective S1PR_1_ agonist, had no effect on the migratory response of BMM (Fig. 2b). Pretreatment with W146, a S1PR_1_ antagonist did not alter S1P-induced BMM migration, either. In contrast, S1P-induced BMM migration was abrogated by JTE-013, a specific S1PR_2_ antagonist or CAY10444, a specific S1PR_3_ antagonist (Fig. 2c). These results manifest that S1PR_2_ and S1PR_3_ are involved in S1P-induced BMM migration.

To further explore the critical role of S1PR_2_ and S1PR_3_, individual S1PR was knocked down by specific siRNA. First we confirmed that S1PR_1_**-**, S1PR_2_**-** or S1PR_3_**-**siRNA effectively silenced the corresponding target gene at the mRNA (Fig. 2d) and protein levels (Fig. 2e). S1PR_1_-, S1PR_2_- or S1PR_3_-targeted siRNA did not alter the expression of other S1PRs, which confirmed the specificity of these siRNAs. Furthermore, silencing S1PR_2_ or S1PR_3_ expression with siRNA abrogated the migration of BMM induced by S1P (Fig. 2f). Altogether, these results suggest that S1P exerts a powerful pro-migratory action on BMM via S1PR_2_ and S1PR_3_.

### G(α)_i/o_, PI3K and Rac1 signals are involved in S1PR_2/3_-mediated BMM migration

**Figure 3:**
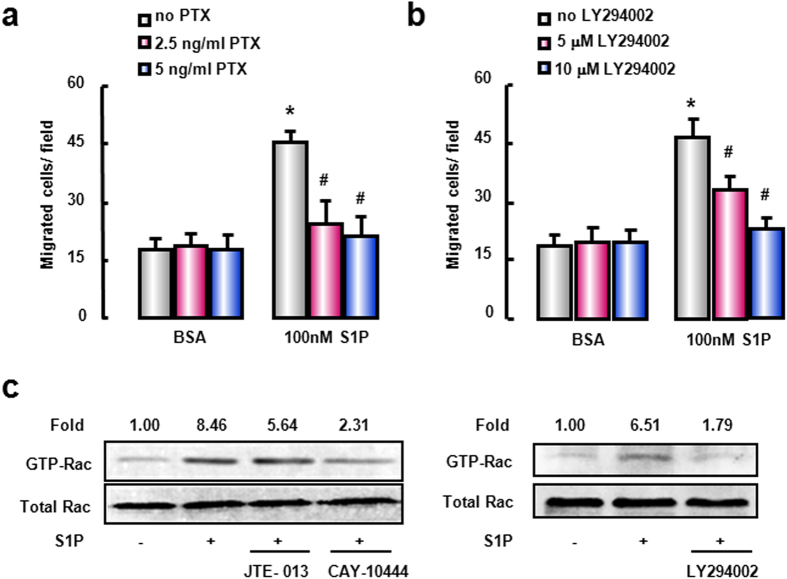
The effect of G(α)_i/o_, PI3K and Rac1 signals on S1P-induced BMM migration. Serum-starved cells were pretreated for 1 hour with G(α)_i/o_ antagonist PTX (**a**) or PI3K antagonist LY294002 (**b**). Pretreated cells were then allowed to migration in the presence of S1P. (**c**) Western blot analysis for activation of Rac1 using active Rac1 Pull-Down and Detection kit. All results were confirmed in three independent experiments at least. *P < 0.05 vs. untreated control cells. ^#^*P* < 0.05 vs. S1P-treated cells alone.

In order to determine the signaling pathways by which S1P exerts its pro-migratory effect on BMM via S1PR_2_ and S1PR_3_, we employed the distinct pharmacological inhibitors of corresponding signaling pathways. S1PRs are G-protein-coupled receptors, and pertussis toxin (PTX, G(α)_i/o_ protein inhibitor) significantly inhibited S1P-induced BMM migration (Fig. 3a). PI3K was found to participate in cell migration of numerous cell types^19^,^25^; therefore we pretreated BMM with LY-294002, a PI3K inhibitor, to examine whether PI3K was involved in S1PR_2/3_-mediated BMM migration, finding that LY-294002 counteracted BMM migration (Fig. 3b). Rac and Rho, members of the small GTPase family, are closely related to cell migration for their molecular switche roles (GTP-bound for active)^26^. For that reason, we first explored whether S1P depended on Rac for its pro-migratory ability. Pull-down analysis displayed that S1P remarkably promoted active GTP-bound Rac1 protein levels in BMM, whereas this added GTP-bound Rac1 conformation by S1P can be inhibited by JTE-013, CAY-10444 or LY294002 (Fig. 3c). By comparison, Rho signal was not involved in this pathway (data not shown). Taken together, these findings indicate that S1P activates G(α)_i/o_-coupled S1PR_2_ and S1PR_3_, then enlarges PI3K and Rac1 signals, finally drives BMM migration.

### Antagonism of S1PR_2_ or S1PR_3_ significantly reduces BMM recruitment in cholestatic liver injury

**Figure 4:**
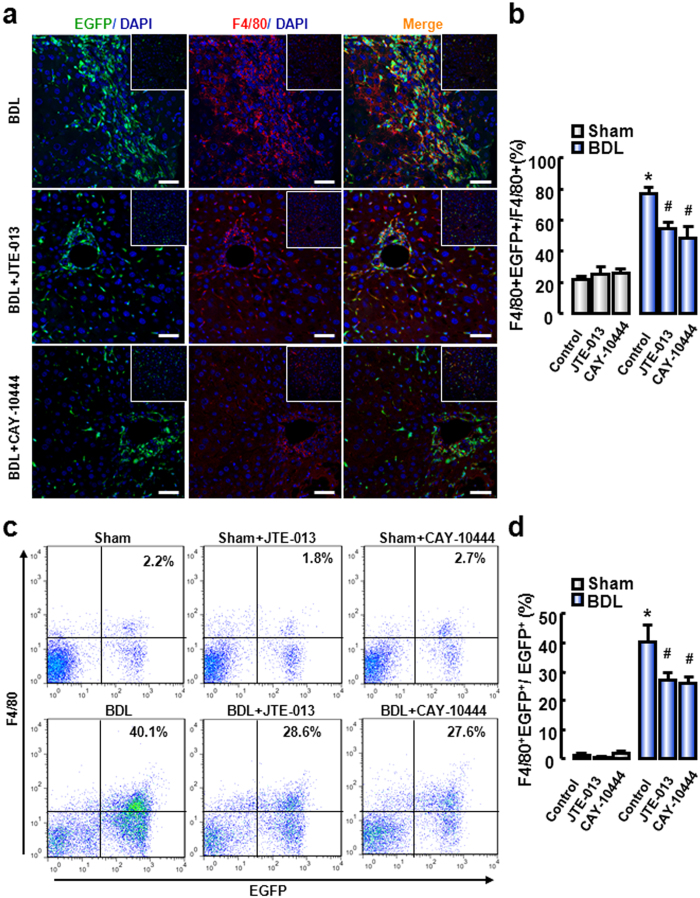
S1PR_2/3_ antagonist reduces BMM recruitment in cholestatic liver injury. (**a**) Representative confocal images to track the macrophages (F4/80^+^, red) of BM origin (EGFP^+^, green) in the BDL-treated livers. DAPI was used to visualize nuclei (blue). Inset: representative images for Sham-treated livers. Scale bars, 50 μm. (**b**) The proportion of BMMs (numbers of cells with yellow color/red color), was measured by Image-Pro Plus. (**c**) Flow-cytometric analysis of the non-paren**c**hymal cells (NPC) in the liver for F4/80. (**d**) The proportion of monocytes/macrophages from BM was measured by FACS Diva 4.1. *P < 0.05 vs. Control. ^#^*P *< 0.05 vs. BDL group (n = 7 per group).

To investigate the potential role of S1PR_2_ and S1PR_3_ in BMM migration during cholestatic liver injury, we performed an EGPF-positive BM cell transplantation experiment followed by BDL-induced liver injury with the administration of JTE-013 or CAY-10444. Then we performed immunofluorescent staining for F4/80, which is a representative marker of mouse macrophages. The results showed that a large amount of EGFP-positive cells in the fibrotic areas were also positive for F4/80, implying that BMM was recruited to the injured liver (Fig. 4a). Strikingly, JTE-013 or CAY-10444 reduced the population of BMM in the fibrotic liver compared with that in BDL-treated mice (Fig. 4b).

To further demonstrate the role of S1PR_2_ and S1PR_3_ in BMM migration in cholestatic liver injury, FACS analysis was performed. After 2 weeks of JTE-013 or CAY-10444 administration, the proportion of BMM (both F4/80^+^ and EGFP^+^) in damaged liver decreased markedly (from 40.1% to 28.6% and 27.6%, respectively) compared with that without JTE-013 or CAY-10444 treatment (Fig. 4c,d). Together, these data confirm that S1PR_2_ and S1PR_3_ play a pivotal role in BMM migration *in vivo*.

### Antagonism of S1PR_2_ or S1PR_3_ attenuates the inflammation of injured liver

**Figure 5:**
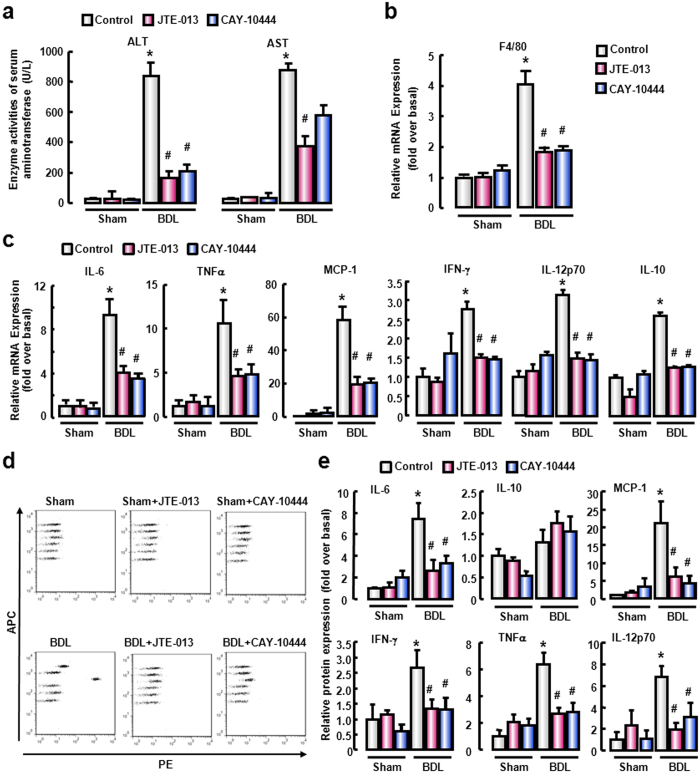
S1PR_2/3_ antagonists attenuate the hepatic inflammation in cholestatic liver injury. (**a**) Serum ALT and AST activity were measured using automatic biochemistry analyzer. (**b**) The mRNA level of F4/80 in liver was measured by real-time RT-PCR. (**c**) The mRNA levels of inflammatory cytokines in liver. Inflammatory cytokines proteins in liver tissues of mice with different treatments were analyzed by BD Cytometric Bead Array. (**d**) Representative scatter plots with six bead populations, which represent IL-6, IL-10, MCP-1, IFN-γ, TNFα and IL-12p70, respectively, based on APC fluorescence intensity from high to low. (**e**) The quantitative results of CBA essay. *P < 0.05 vs. Control. ^#^*P *< 0.05 vs. BDL group (n = 7 per group).

Given the effect of S1P/S1PR signaling on BMM migration and the role of macrophages in inflammation responses, next we assessed the potential impact of S1PR_2_ and S1PR_3_ antagonists on liver inflammation. Biochemical parameters represented by serum aspartate aminotransferase (AST) and alanine aminotransferase (ALT) were alleviated after administration of JTE-013 or CAY-10444 (Fig. 5a). Besides, there was a severe reduction in the mRNA level of macrophage marker F4/80 (Fig. 5b).

Given the importance of inflammatory cytokines and chemokines in inflammatory responses, next we assessed the effect of S1PR_2_ and S1PR_3_ antagonists on inflammatory cytokines and chemokines, including pro-inflammatory cytokine tumor necrosis factor-α (TNF-α), interferon-γ (INF-γ) and interleukin-12p70 (IL-12p70), both pro- and anti-inflammatory cytokine interleukin-6 (IL-6), anti-inflammatory cytokine IL-10, and chemokine monocyte chemoattractant protein-1 (MCP-1). JTE-013 or CAY-10444 administration obviously decreased the mRNA and protein levels of inflammatory cytokines in BDL-treated damaged liver (Fig. 5c–e). These results indicated that antagonism of S1PR_2_ or S1PR_3_ indeed relieved the extent of inflammation in mice of BDL models.

In order to confirm our conclusion, we adopted an established model in which chemically modified and stable siRNAs of S1PR_2_ and S1PR_3_ were injected rapidly into the tail vein, using a “hydrodynamic transfection method”. S1PR_2_**-** or S1PR_3_**-**siRNA successfully down-regulated each mRNA level by 60.5% and 42.5% in BM (Supplementary Fig. 1), with no alteration on the expression of other S1PRs, which confirmed the effectiveness and specificity of these siRNAs. Moreover, FACS analysis for F4/80 showed that in BDL-induced damaged liver the proportion of EGFP^+^/F4/80^+^ cells (indicating they were BMM) among nonparenchymal cells increased markedly compared with that in Sham liver. However, S1PR_2_ or S1PR_3_ siRNA administration markedly decreased the number of BMM in BDL mice (Supplementary Fig. 2). These results further confirmed the contribution of S1PR_2_ and S1PR_3_ to BMM migration *in vivo*.

### Antagonism of S1PR_2_ or S1PR_3_ attenuates BDL-induced liver fibrosis

**Figure 6:**
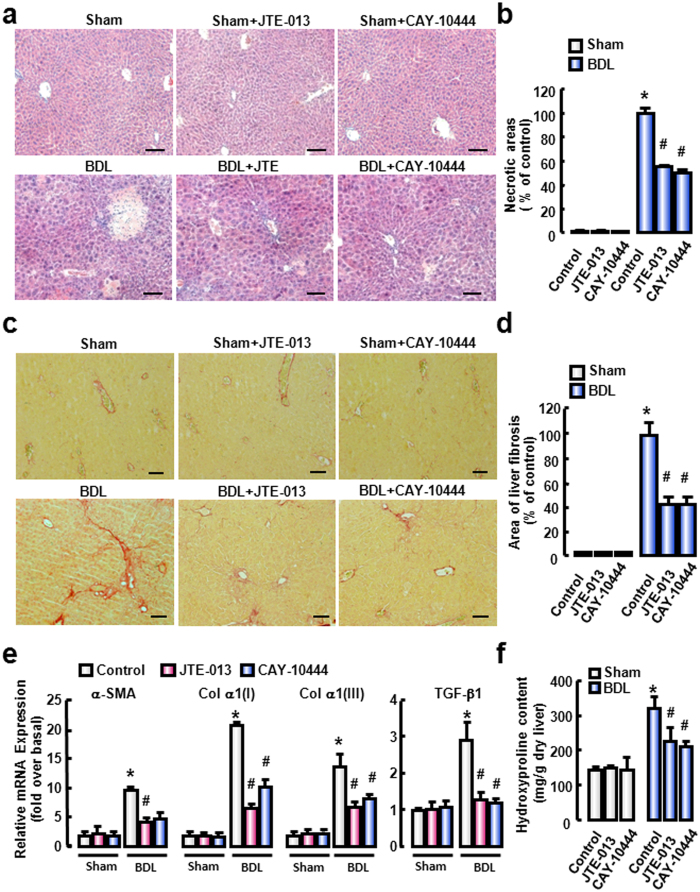
S1PR_2/3_ antagonists attenuate the hepatic fibrosis in cholestatic liver injury. (**a**) Representative H&E-staining liver sections after 2-week BDL with JTE-013/CAY-10444 administration. Scale bars, 50 μm. (**b**) Quantification of necrotic areas. (**c**) Representative images of Sirius Red staining in the BDL-treated livers with JTE-013/CAY-10444 administration. Scale bars, 50 μm. (**d**) Quantitative analysis of liver fibrosis. (**e**) Relative mRNA levels of hepatic *α-SMA, Col α1(I), Col α1(III)* and *TGF-β1*. (**f**) Hydroxyproline content in liver. *P < 0.05 vs. Control. ^#^*P* < 0.05 vs. BDL group (n *=* 7 per group).

Finally, we analyzed the effects of S1PR_2_ and S1PR_3_ antagonists on the underlying liver fibrosis process. H&E-stained sections showed a significant decrease in liver injury following JTE-013 or CAY-10444 administration in BDL-treated mice (Fig. 6a). Inflammation area quantified by digital image analysis was dramatically reduced after JTE-013 or CAY-10444 administration (Fig. 6b). Hepatic collagen deposition was evaluated by morphometric analysis of Sirius red staining and quantified by digital image analysis. The result reviewed that collagen deposition was markedly attenuated after the administration of JTE-013 or CAY-10444 (Fig. 6c,d). Furthermore, we examined the effect of JTE-013 or CAY-10444 on fibrosis markers, including α-smooth muscle actin (α-SMA), procollagen α1(I) (Col α1(I)), procollagen α1(III) (Col α1(III)) and TGF-β1 in BDL-induced mouse liver fibrosis. After 2 weeks BDL, *α-SMA, Col α1(I), Col α1(III)* and *TGF-β1* mRNA expressions in liver tissue were distinctly augmented. However, JTE-013 or CAY-10444 administration presented a significant drop in the mRNA expression of these fibrotic markers compared with that in BDL-treated mice (Fig. 6e). Moreover, we also measured the total liver hydroxyproline content (Fig. 6f). After JTE-013 or CAY-10444 administration, there was a significant decrease in hydroxyproline content compared with that in BDL-treated mice. These results demonstrate that antagonism of S1PR_2_ or S1PR_3_ attenuates BDL-induced liver fibrosis.

## Discussion

**Figure 7:**
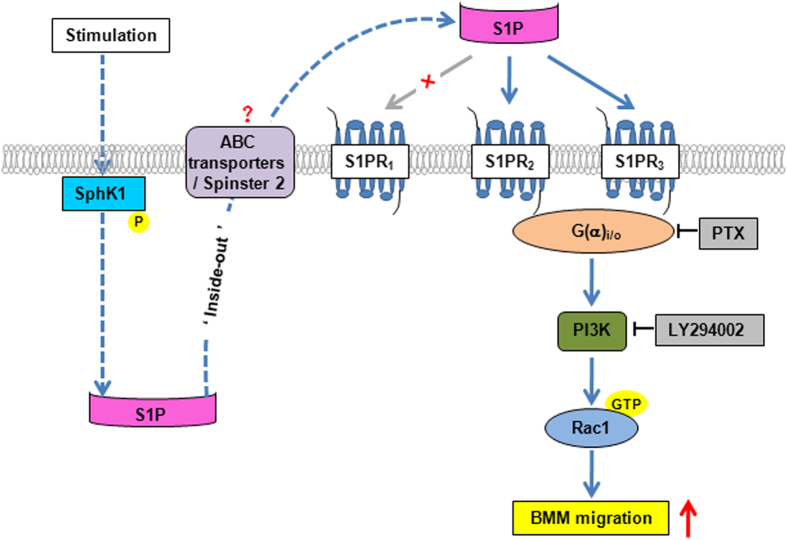
Scheme of BMM migration induced by S1P/S1PR_2/3_. S1P exerts a powerful migratory effect on BMMs via S1PR_2_ and S1PR_3_, which involves the activation of G(α)_i/o_, PI3K and Rac1 signaling pathways.

In the current study, we identify S1PR_2/3_ as a critical regulator of BMM recruitment to the sites of liver injury for the first time. *In vitro* S1P exerts a powerful migratory effect on BMMs via S1PR_2_ and S1PR_3_, which involves G(α)_i/o_, PI3K and Rac1 signaling pathways. *In vivo*, administration of S1PR_2/3_ antagonist to BDL-treated mice significantly reduces BMM recruitment in the fibrotic liver, decreases the generation of inflammatory cytokines, and relieves the extent of inflammation and fibrosis in cholestatic liver injury. A concept diagram is drawn to summarize our major findings (Fig. 7).

Recently, a potential role of BM-derived cells in liver fibrosis is gaining attention^8^,^27^,^28^. There are a number of studies reporting that BM-derived cells can migrate to the injured liver, transdifferentiate into hepatic myofibroblasts and ultimately participate in the progress of liver fibrosis^29^,^30^. Among the BM-derived cells, we have been focusing on BMMs in this study, and have demonstrated that BMMs are recruited to the insulted liver after BDL. In general, migration of BMMs to liver is an essential step in the supplemental update of Kupffer cells; however, excessive or prolonged influx of BMM is pathological response such as liver inflammation and fibrosis^31^,^32^,^33^. Among the plethora of growth factors that regulate cell migration, S1P is probably one of the most potent mediators. The previous studies of our lab have proved that S1P mediates the homing of BM-derived mesenchymal stem cells in liver fibrogenesis. Here, we examine the pro-migratory activity of S1P in BMMs, and show that S1P exhibits a profound effect on BMM migration both *in vitro* and in cholestatic liver injury.

Most of the characterized actions of S1P are mediated through its G protein-coupled receptors S1PRs^34^. S1PR_1_ is coupled exclusively to G_i_, G_q_ whereas S1PR_2_ and S1PR_3_ are coupled to G_i_, G_q_, and G_12/13_^35^. S1PR subtypes are expressed in distinct combinations in different cell types to produce an appropriate biological action. From the pattern of expression, it is likely to understand the pathophysiological role of each S1PR^36^. In the present study, we show that S1PR_1_, S1PR_2_ and S1PR_3_ are all clearly detected in BMMs, as determined by RT-PCR and Western blot analysis. By employing various S1PR agonist (SEW2871), antagonists (W146, JTE-013, CAY-10444), or silencing S1PR expression by cell transfection with specific siRNAs, we provide evidence that S1P-induced BMM migration requires the expression of S1PR_2_ and S1PR_3_. There are some reports on the role of S1PR_1_ in cell migration as well. For example, apoptotic cells enhance human macrophage migratory potential through regulating S1PR_1_ expression^37^. In addition, S1PR_1_-selective agonist CYM inhibits acute graft-versus-host disease by reducing macrophage infiltration^38^. The reason for such a discrepancy may come from the use of different cell types in different diseases. However, there is some evidence on the role of S1PR_2_ and S1PR_3_ in cell migration. A recent study has indicated that suppression of the S1PR_2_ function by either siRNA or S1PR_2_ antagonist (JTE-013) completely blocked S1P-mediated cell migration and invasion in B16 melanoma^39^. In another report, S1PR_2_ mediated the migration and chemotaxis in cultured human umbilical vein endothelial cells^40^. Our previous study has also reported that S1PR_3_ are implicated in the migration of BM-derived mesenchymal stem cells from BM to damaged liver^29^. All together, these results supported the role of S1PR_2_ and S1PR_3_ in cell migration. The reason why both S1PR_2_ and S1PR_3_ are required to stimulate BMM migration in our current study may lie in that they can bind to and activate the same G protein (G(α)_i/o_), which leads to activation of downstream signaling pathways, such as PI3K and Rac. Further studies will be needed to evaluate the effects of S1PR_2/3_ on other downstream second messenger molecules, such as Ca^2+^ influx and protein kinase C, and their upstream/downstream relationships. In fact, we measured the activation of PKCα by S1P by Western blot. The results showed that S1P could activate PKCα, which can be blocked by S1PR_2_ or S1PR_3_ inhibitor/ siRNA (data not shown).

Members of the Rho family of small guanosine triphosphatases (GTPases), such as Rac, Rho and Cdc42, regulate the organization of the actin cytoskeleton, thus are implicated in the modulation of cell migration. Rho controls the assembly of actin stress fibers and focal adhesion complexes; Rac regulates actin filament accumulation at the plasma membrane to produce lamellipodia and membrane ruffles; and Cdc42 stimulates the formation of filopodia^41^. Here we show that S1P depended on Rac1, rather than Rho, for its pro-migratory ability. Pull-down analysis reveals that the remarkably added GTP-bound Rac1 conformation by S1P in BMMs is inhibited by JTE-013 or CAY-10444. Beside Rho and Rac, a large number of studies have demonstrated that PI3K activity is also necessary and sufficient for cell migration^42^. Therefore, we next focus on the effect of PI3K on S1P-induced BMM migration and the relationship between PI3K and Rac. A PI3K inhibitor, LY-294002, apparently inhibits S1P-mediated BMM migration, and results in a decreased expression of active Rac in cells treated by S1P. The results above imply that PI3K may function as an upstream activator of Rac. Further studies will be needed to illustrate the exact molecular mechanisms by which S1P contributes to BMM migration in liver injury.

In summary, our findings present evidence that S1P/S1PR_2/3_ axis is an important regulator of BMM motility *in vitro* and *in vivo*. S1P is able to activate its receptors S1PR_2_ and S1PR_3_, and then enlarges PI3K and Rac signaling pathways, at last drives the migration and recruitment of BMMs. Our results provide new compelling information on the role of S1PR_2/3_ in liver injury and opens new perspectives for the pharmacological treatment of hepatic fibrosis.

## Methods

### Materials

1640 was from Invitrogen (Grand Island, NY). Fetal bovine serum was from Biochrom (Berlin, Germany). PCR reagents were from Applied Biosystems (Foster City, CA). S1P and dihydro-S1P (H_2_S1P) were from Biomol (Tebu, France). JTE-013 and CAY-10444 were from Cayman Chemical (Ann Arbor, MI). Anti-F4/80 antibody used for flow cytometry analysis was from BD Biosciences (San Jose, CA). Histopaque-1077 and other common reagents were from Sigma (St. Louis, MO).

### Isolation and cultivation of mice BMMs

Cells were obtained from the tibia and femur bone marrow of ICR mice (aged three weeks) and were cultured in the presence of L-929 conditioned medium as described in detail previously^8^. The identification of BMMs is assessed using immunocytochemistry analysis of F4/80 expression. All animal works were carried out in accordance with the approved guidelines of the Ethics Committee of Capital Medical University.

### Mouse models

ICR mice aged 6 weeks received lethal irradiation (8 Grays), and immediately received transplantation by a tail vein injection of 1.5 × 10^7^ whole BM cells which obtained from 3-week-old EGFP transgenic mice. Four weeks later, BM was reconstructed and the chimera mice with EGFP-labeled BM cells were subjected to the BDL-induced liver injury.

ICR mice were allocated randomly to two experimental groups, either BDL or sham operations were performed as described previously^29^,^30^. In brief, mice were anesthetized to receive a midline laparotomy, and then the common bile duct was exposed and ligated three times. Two ligatures were placed in the proximal portion of the bile duct, and one ligature was located in the distal portion of the bile duct. The bile duct was then cut between the ligatures. The abdomen was closed in layers, and mice were allowed to recover on a heat pad. Two weeks later, mice were anesthetized to collect blood and liver samples.

10 mg/kg B.W. JTE-013 or CAY10444 was administered intraperitoneally one day before BDL operation, and twice per week after BDL operation for two weeks (n =7 per group). After two weeks, mice were sacrificed and liver tissue was harvested (n = 7 per group). All animal works were carried out in accordance with the approved guidelines of the Ethics Committee of Capital Medical University.

### Immunofluorescence staining

Liver samples were fixed in 4% paraformaldehyde and embedded in Tissue Tek OCT compound (Electron Microscopy Sciences, Japan). Six micrometers of frozen section were used for immunofluorescence. They were blocked with 3% BSA, then incubated with anti-F4/80 rat monoclonal antibody (1:100, Santa Cruz Biotechnology, CA) followed by secondary antibody conjugated with Cy3 (1:100, Jackson Immuno-Research, West Grove, PA). The sections were covered with Vectashield mounting medium containing DAPI and observed under confocal microscope (LSM510, Carl Zeiss MicroImaging GmbH, Germany).

### Fluorescence-activated cell sorting (FACS)

Non-parenchymal cells of mouse liver were isolated as described previously^8^. APC-F4/80 antibody (eBioscience, San Diego, CA) and its isotype-matched negative control were added to the non-parenchymal cells, respectively. After 15min incubation in the dark, the cells were washed with PBS and subjected to FACS, which was performed on a FACSAria and analysed with FACS Diva 4.1 (BD, Biosciences).

### Measurement of cytokines and chemokine by cytometric bead array (CBA)

Liver tissues (40 mg) were homogenized and lysed in 30 μL lysis buffer. IL-6, IL-10, MCP-1, IFNγ, TNF and IL-12p70 in liver homogenates were measured using BD CBA Mouse Inflammation Kit (Catalog No.552364) and FACS.

### Real-time RT-PCR

Extraction of total RNA from liver frozen specimen and Real-time RT-PCR was performed in an ABI Prism 7300 sequence detecting system (Applied Biosystems), as described previously. Primers were as follows: Mouse *α-SMA*: sense, 5′-ATGCTCCCAGGGCTGTTTT-3′; antisense, 5′-TTCCAACCATTACTCCCTGATGT-3′. *Col α1(I)*: sense, 5′-AGGGCGAGTGCTGTGCTTT-3′; antisense, 5′-CCCTCGACTCCTACATCTTCTGA-3′. *Col α1(III)*: sense, 5′-TGAAACCCCAGCAAAACAAAA-3′; antisense, 5′-TCACTTGCACTGGTTGATAAGATTAA-3′. *TNFα*: sense, 5′-GGCAGGTTCTGTCCCTTTCA-3′; antisense, 5′-CTGTGCTCATGGTGTCTTTTCTG-3′. *MCP-1*: sense, 5′-TCTGGGCCTGCTGTTCACA-3′; antisense, 5′-GGATCATCTTGCTGGTGAATGA-3′. *TGF-β1*: sense, 5′-TGCGCTTGCAGAGATTAAAA-3′; antisense, 5′-CTGCCGTACAACTCCAGTGA-3′. *IL-6*: sense, 5′-CTCTGGGAAATCGTGGAAATG-3′; antisense, 5′-AAGTGCATCATCGTTGTTCATACA-3′. *F4/80*: sense, 5′-AGCACATCCAGCCAAAGCA-3′; antisense, 5′-CCATCTCCCATCCTCCACAT-3′. *IL-10*: sense, 5′-CAGTACAGCCGGGAAGACAATAA-3′; antisense, 5′- CCGCAGCTCTAGGAGCATGT-3′. *IFN-γ*: sense, 5′- TCTGAGACAATGAACGCTACACACT-3′; antisense, 5′- TGGCAGTAACAGCCAGAAACA-3′. *IL-12p70*: sense, 5′- CCTGAAGTGTGAAGCACCAAATT-3′; antisense, 5′- CTTCAAGTCCATGTTTCTTTGCA-3′. *18S rRNA*: sense, 5′-GTAACCCGTTGAACCCCATT-3′; antisense, 5′-CCATCCAATCGGTAGTAGCG-3′.

### Western blot analysis

Western blot analysis of S1PR_1–3_ were performed with 50 μg of protein extract, obtained as described previously, using rabbit polyclonal antibody to S1PR_1–3_ (1:500, Santa Cruz Biotechnology, Santa Cruz, CA) and the appropriate IRDyeTM 800-conjugated secondary antibody (1:10,000). Signals were detected using the Odyssey Imaging System (LI-COR Biosciences, Lincoln, NE) and analyzed with Odyssey software. Results were normalized relative to the GAPDH (rabbit antibody, 1:1000; Sigma, St. Louis, MO) or tubulin (rabbit antibody, 1:1000, Epitomics, Burlingame, CA) expression to correct for variations in protein loading and transfer.

### Hydroxyproline content assay

Hydroxyproline content of the liver was measured as previously described^24^.

### Quantitative Analysis of Liver Fibrosis

The fibrotic or necrotic areas were assessed as previously described^43^. The mean value of 15 randomly selected areas per sample was used as the expressed percentage of fibrotic or necrotic area.

### Migration assay

Cell migration was determined in Boyden chambers as described previously. In brief, serum-starved macrophages were seeded to the upper chamber and were exposed to JTE-013, CAY-10444 or vehicle (negative control) for 1 hour. Various concentrations of S1P or H_2_S1P were added to the lower chamber. The chambers were incubated for 4 hours at 37 °C in 5% CO_2_. Cells migrated to the lower surface of the filter were stained and quantified by cell counting.

### Active Rac1 Pull-Down and Detection

Active Rac1 were extracted using pull-down and detection kit (Catalog No 16118, Thermo Scientific, Lafayette, CO). Active and total Rac were showed by Western blot.

### RNA interference

siRNA sequence targeting specifically mouse S1PR_1–3_ was purchased (L-051684-00, L-063765-00, L-040959-00; Thermo Scientific, Lafayette, CO). Forty to 50% percent confluent BMMs were prepared. Transient transfection of siRNA (40 nM) was preformed by using Lipofectamine RNAiMAX (Invitrogen, Carlsbad, CA) as recommended by the manufacturer. Control cells were treated with 40 nM RNAi Negative Control Duplexes (scramble siRNA). After 48 hours, cells were used to perform further experiment.

### Statistical analysis

The results are expressed as mean ± SEM. Statistical significance was determined by Student’s t-test or ANOVA. *P *< 0.05 was considered significant.

## Additional Information

**How to cite this article**: Yang, L. *et al.* Sphingosine 1-Phosphate Receptor 2 and 3 Mediate Bone Marrow-Derived Monocyte/Macrophage Motility in Cholestatic Liver Injury in Mice. *Sci. Rep.* **5**, 13423; doi: 10.1038/srep13423 (2015).

## Supplementary Material

Supplementary Information
