# Supplementary material for: Sphingosine 1-Phosphate Receptor 2 and 3 Mediate Bone Marrow-Derived Monocyte/Macrophage Motility in Cholestatic Liver Injury in Mice
Source: Sci Rep. 2015 Sep 1;5:13423. doi: 10.1038/srep13423 (PMC4555045; doi:10.1038/srep13423)

Sphingosine 1-Phosphate Receptor 2 and 3 Mediate Bone Marrow-Derived Monocyte/Macrophage Motility in Cholestatic Liver Injury in Mice

Le Yang[1], Zhen Han[1], Lei Tian, Ping Mai, Yuanyuan Zhang, Lin Wang and Liying Li*

Supplementary Information

Supplementary Methods

siRNA treatment in vivo

Chemically modified and stable siRNAs of $S1PR_2$ (sense, UAA CUC CCG UGC AGU GGU UUU; antisense, 5'-pAAC CAC UGC ACG GGA GUU AUU) and $S1PR_3$ (sense, GGA GGG CAG UAU GUU CGU AUU; antisense, 5'-pUAC GAA CAU ACU GCC CUC CUU) were purchased from Thermo Scientific (Lafayette, CO), and were delivered *in vivo* using a "hydrodynamic transfection method", by which 50 μg siRNA dissolved in 1 mL PBS was rapidly injected into the tail vein. Control mice were injected with an equal volume of scramble siRNA dissolved in PBS. These siRNAs were injected one day before BDL-induced liver injury, and twice per week after BDL operation for 2 weeks.

Supplementary Figure legends

Supplemental Figure 1. The effect of $S1PR_2$- or $S1PR_3$-siRNA *in vivo* in cholestatic liver injury. Knock-down of $S1PR_2$ or $S1PR_3$ by their siRNAs *in vivo* in BM. * $p < 0.05$ vs. Controls (n = 7 per group).

Supplementary Figure 2. $S1PR_2$ or $S1PR_3$ siRNA *in vivo* reduced the recruitment of BMM. The proportion of BMM ($EGFP^+/F4/80^+$) among nonparenchymal cells were analyzed by FACS (n = 7 per group).

supplementary figure 1

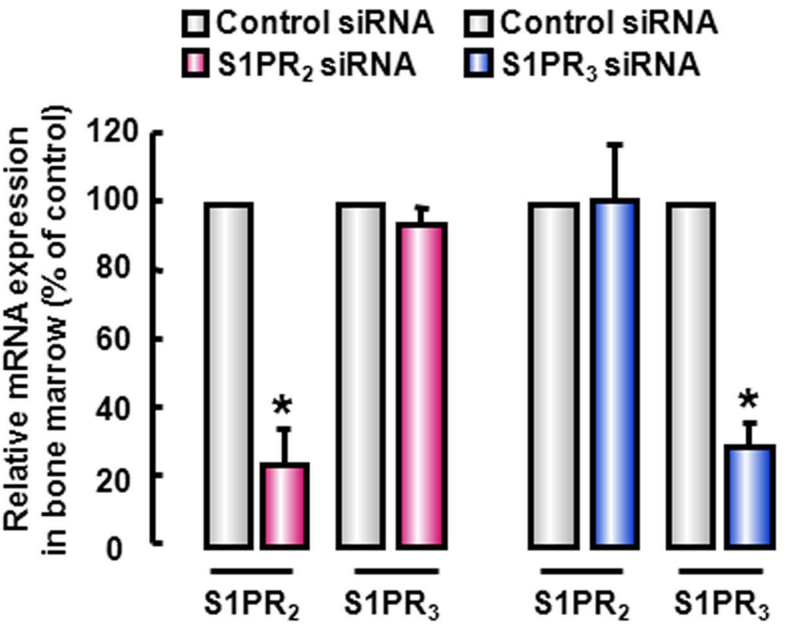

supplementary figure 2

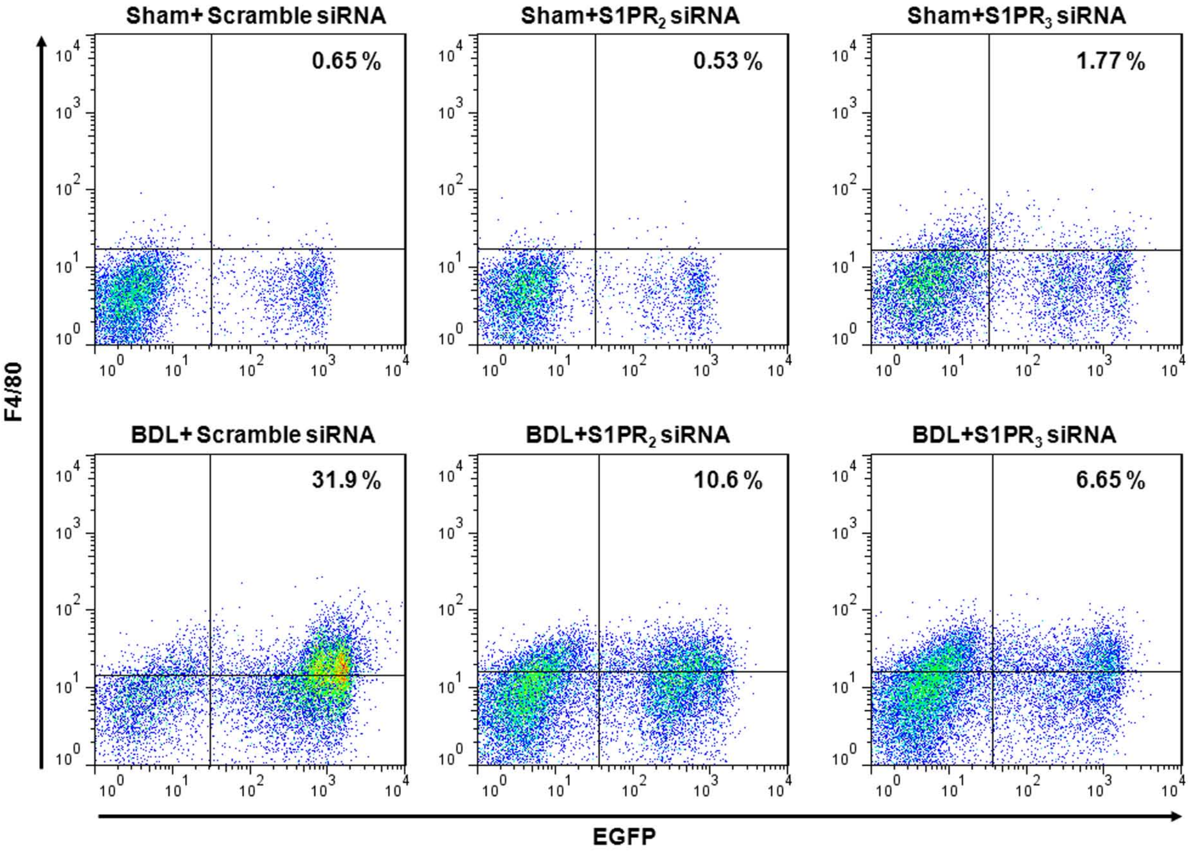

Supplement: Supplementary Information [file srep13423-s1.pdf]
